# Chronic fatigue syndromes: real illnesses that people can recover from

**DOI:** 10.1080/02813432.2023.2235609

**Published:** 2023-11-29

**Authors:** The Oslo Chronic Fatigue Consortium, Tomas Nordheim Alme, Anna Andreasson, Tarjei Tørre Asprusten, Anne Karen Bakken, Michael BJ Beadsworth, Birgitte Boye, Per Alf Brodal, Elias Myrstad Brodwall, Kjetil Gundro Brurberg, Ingrid Bugge, Trudie Chalder, Reidar Due, Hege Randi Eriksen, Per Klausen Fink, Signe Agnes Flottorp, Egil Andreas Fors, Bård Fossli Jensen, Hans Petter Fundingsrud, Paul Garner, Lise Beier Havdal, Helene Helgeland, Henrik Børsting Jacobsen, Georg Espolin Johnson, Martin Jonsjö, Hans Knoop, Live Landmark, Gunvor Launes, Mats Lekander, Hannah Linnros, Elin Lindsäter, Helena Liira, Lina Linnestad, Jon Håvard Loge, Peter Solvoll Lyby, Sadaf Malik, Ulrik Fredrik Malt, Trygve Moe, Anna-Karin Norlin, Maria Pedersen, Siv Elin Pignatiello, Charlotte Ulrikka Rask, Silje Endresen Reme, Gisle Roksund, Markku Sainio, Michael Sharpe, Ruth Foseide Thorkildsen, Betty van Roy, Per Olav Vandvik, Henrik Vogt, Hedda Bratholm Wyller, Vegard Bruun Bratholm Wyller

**Affiliations:** a Department of Paediatrics and Adolescent Medicine, Akershus University Hospital, Lørenskog, Norway; b Department of Clinical Epidemiology, Karolinska Institutet, Stockholm, Sweden; c General Practitioner, Hjelmeland, Norway; d VID Specialized University, Oslo, Norway; ba St. Olavs Hospital, Trondheim, Norway; e Tropical and infectious Disease Unit, Royal Liverpool University Hospital. Liverpool University Hospitals Foundation Trust, Liverpool, UK; f Department of Behavioral Medicine, University of Oslo, Oslo, Norway; g Institute of Basic Medical Sciences, University of Oslo, Oslo, Norway; h Institute of Clinical Medicine, University of Oslo, Oslo, Norway; i Division for Health Services, Norwegian Institute of Public Health, Oslo, Norway; j Department of Child and Adolescent Mental Health in Hospitals, Oslo University Hospital, Oslo, Norway; k Department of Psychological Medicine, Institute of Psychiatry, Psychology and Neuroscience, King’s College London, London, England; m Department of Sport, Food and Natural Sciences, Western Norway University of Applied Sciences, Bergen, Norway; n Research Clinic for Functional Disorders and Psychosomatics. Aarhus University Hospital, Aarhus, Denmark; o Centre for Epidemic Interventions Research, Norwegian Institute of Public Health, Oslo, Norway; bb Department of General Practice, University of Oslo, Oslo, Norway; p Faculty of Medicine and Health Sciences, Department of Public Health and Nursing, General Practitioner Research Unit, Norwegian University of Science and Technology (NTNU), Trondheim, Norway; q Nydalen Helsehus, Oslo, Norway; r The Child & Adolescent Health Services, University Hospital of North Norway, Tromsø, Norway; s Department of Clinical Sciences, Liverpool School of Tropical Medicine, Liverpool, England; t Department of Behavior Medicine, Karolinskal University Hospital, Stockholm, Sweden; u National Advisory Unit on Psychosomatic Disorders in Children and Adolescents. Department of Child and Adolescent Mental Health in Hospitals, Oslo University Hospital, Oslo, Norway; v Department of Pain management and research, Oslo University Hospital, Oslo, Norway; bd CatoSenteret Rehabilitation Center, Son, Norway; w University of Oslo, Oslo, Norway; x Department of Clinical Neuroscience, Karolinska Institutet Stockholm, Sweden; y Department of Medical Psychology, Amsterdam University Medical Centres, Location University of Amsterdam, Amsterdam, The Netherlands; z Department of Psychology, NTNU, Trondheim, Norway; aa University of Bergen, Bergen, Norway; ab Stress Research Institute, Department of psychology, Stockholm University, Division of Psychology/Osher Center for Integrative Health, Department of Clinical Neuroscience, Karolinska Institutet, Stockholm, Sweden; ac Department of Health, Medicine, and Caring Sciences Pain and Rehabilitation Center, Linköping University Hospital, Linköping, Sweden; ad Center for Psychiatry Research, Department of Clinical Neuroscience, Karolinska Institutet, Stockholm, Sweden; ae Helsinki University Hospital, Helsinki, Finland; af Saglia medical center, Vestby, Norway; aj Division of Clinical Neuroscience. Institute of Clinical Medicine, University of Oslo, Oslo, Norway; ak Chief Physician, Falck Norge, Oslo, Norway; an Unit Psychosomatic medicine and CL psychiatry, Rikshospitalet, Oslo University Hospital, Oslo, Norway; ao Department of Child and Adolescent Psychiatry, Aarhus University Hospital, Aarhus, Denmark; ap The Mind Body Lab, Department of Psychology, University of Oslo; aq General Practitioner, Klosterhagen Legesenter, Norway; ar Outpatient Clinic for Functional Disorders, Helsinki University Hospital, Helsinki, Finland; as Psychological Medicine, University of Oxford, Oxford, UK; at Department of Medicine, Diakonhjemmet Hospital, Oslo, Norway; av Department of Medicine, Lovisenberg Diaconal Hospital, Oslo, Norway; aw Department of Community Medicine and Global Health, Institute for Health and Society, Faculty of Medicine, University of Oslo, Oslo, Norway; ax Clinical Psychologist, Oslo, Norway

**Keywords:** Chronic fatigue syndrome, Myalgic encephalomyelitis, Long Covid, Chronic illness narrative, Multidimensional explanations, Rehabilitation strategies, Patient-centered care

## Abstract

The ‘Oslo Chronic Fatigue Consortium’ consists of researchers and clinicians who question the current narrative that chronic fatigue syndromes, including post-covid conditions, are incurable diseases. Instead, we propose an alternative view, based on research, which offers more hope to patients. Whilst we regard the symptoms of these conditions as real, we propose that they are more likely to reflect the brain's response to a range of biological, psychological, and social factors, rather than a specific disease process. Possible causes include persistent activation of the neurobiological stress response, accompanied by associated changes in immunological, hormonal, cognitive and behavioural domains. We further propose that the symptoms are more likely to persist if they are perceived as threatening, and all activities that are perceived to worsen them are avoided. We also question the idea that the best way to cope with the illness is by prolonged rest, social isolation, and sensory deprivation.Instead, we propose that recovery is often possible if patients are helped to adopt a less threatening understanding of their symptoms and are supported in a gradual return to normal activities. Finally, we call for a much more open and constructive dialogue about these conditions. This dialogue should include a wider range of views, including those of patients who have recovered from them.

## The current public narrative

Severe, persistent fatigue conditions are a pressing public health issue commonly encountered in primary care settings. There is an increasingly dominant public narrative that these fatigue conditions are best understood as chronic and incurable multisystem diseases, often coupled with the prediction that patients cannot recover and that activity is harmful [1–3]. This narrative is most commonly expressed by campaigners concerned with chronic fatigue syndrome (CFS)/myalgic encephalomyelitis (ME) [4], but more recently by those writing about post-covid-19 condition [5], often referred to as Long Covid. As a Consortium of researchers, academics, and clinicians interested in the causes and treatments of fatigue and fatigue related conditions, as well as representatives of patients who have suffered from these illnesses themselves, we propose that a different narrative also needs to be heard. This alternative narrative is based on scientific evidence and offers patients a realistic hope of improvement and recovery.

## Current diagnostic labels are of limited value

Diagnoses guide treatment. However, although many of the diagnoses given to patients with these fatigue related conditions, such as CFS/ME, post-covid-19 conditions and burnout, have questionable validity and reliability [1,6–8], and indeed overlap [9], they imply quite different treatments. Persistent fatigue also occurs in many other illnesses [7,10–12] and is therefore unlikely to indicate a distinct illness with specific pathology [13]. Furthermore, post-exertional malaise (PEM), thought by some to be specific to CFS/ME, also occurs in patients with other diagnoses [1]. Given this lack of evidence for the specificity of the diagnosis of CFS/ME [14], it is surprising that it has often been portrayed as a distinct disease requiring treatment different to that from other similar conditions (for example [1,2]).

## It is time for another perspective

After 40 years of research into CFS/ME [15] neither a specific biological defect or pathology, nor a specific biomarker, has been identified. Whilst many pathophysiological abnormalities have been reported, these remain as non-specific associations. Similar abnormalities have also been found in patients with other illnesses including chronic pain and fibromyalgia [16,17], as well as in illnesses conventionally referred to as ‘psychological’ [18–20]. We therefore think it is time to explore alternative perspectives that include psychological, social as well as biological factors [21].

## Symptoms are both real and generated by the brain

This new perspective views the symptoms of these fatigue related conditions as real. These symptoms, like all perceptions, arise from synchronized activity of complex neural networks in the brain. Whilst such activity may be driven by signals arising in the tissues of the body, it may occur without such signals [22]. The experience of pain, for example, can arise from expectations based on prior experience, without any neuronal input from peripheral sense organs and influenced by the interplay of biological, psychological, and social factors [23–26].

## These conditions can be explained

Research from several fields including neuroscience, evolutionary biology, and physiology provides promising explanations for understanding symptom onset, development, and persistence. Faced by perceived threats to our wellbeing, our brain networks generate alarms in the form of symptoms, such as fatigue and pain, to warn us and shut us down. These alarms may be seen as crucial processes selected through evolution to keep us safe. More specifically, pain signals tissue damage, and fatigue signals a disbalance between effort needed, expected reward and available resources [27], but they are also regulated and influenced by context. These perceived threats to our safety can evoke a stress response of automated bodily defense mechanisms consisting of interlinked immunological, hormonal, cognitive, and behavioral adjustments. This response is initially temporary and adaptive, but may become persistent and maladaptive chronically affecting sleep and cognitive functioning [28,29]. A high level of neuroplasticity in this alarm system risks associative learning, whereby the alarms are reactivated by innocent cues (by classical conditioning). The way we experience a situation is strongly influenced by our previous experience and expectations [22,30,31]. Other processes that contribute to symptom persistence include unconscious bias in attention and interpretation, sensitization to stimuli and changes in perception effort [32].

## Activity as well as rest is needed for rehabilitation

Building on this understanding, the presence of fatigue and other symptoms after activity does not necessarily mean that such activity is dangerous or that there is ‘lack of energy in the body’. Rest is beneficial after acute stressors, such as an infection, but a gradual and controlled approach to increasing activity is crucial for rehabilitation. Patients should feel secure and in control throughout the process. Such rehabilitation can reduce symptoms by allowing the systems described above to readapt to activity [33], and can be readily delivered in primary care. By contrast, the approach often recommended by the public narrative of inactivity, isolation, and sensory deprivation, risks worsening symptoms and associated disability [34–36].

Patients do recover and get back to work [37,38], and patients can get help that improves their chances of recovery [39]. Giving them credible and positive explanations of their symptoms gives them hope that they will be able to return to valued activities [40,41]. Individualized rehabilitative treatments such as cognitive behavioral therapy (CBT) and graded exercise therapy (GET), based on this understanding and given within a supportive therapeutic relationship, help patients to gain control of their illness [42]. When given correctly by appropriately trained therapists these treatments can offer useful improvement for many and recovery for some [43].

## We need to explore all avenues that may lead to effective treatments

We are well aware that some patients have met mistrust and even dismissal of their suffering and may also feel that their illness is stigmatized. These experiences are regrettable, should not have happened and can result in patients feeling scared and angry. We are also aware that some feel that this dismissal justifies a defensive narrative that decries the involvement of psychological and social factors and promotes the idea of an ‘incurable physical disease’. In order to maintain the dominance of this narrative any patients, clinicians, and researchers who question it may be attacked, harassed and unfairly charged with conspiring against vulnerable patients [44]. We believe such actions to be unethical and incompatible with good science and properly informed patient care.

## The patient voice is important and must include the recovered

We do believe that the patients´ voice is important and that this voice must include those who improved or recovered from the illness, and not just those who remain ill [45,46]. Those who have improved or recovered through cognitive, behavioural, and stress-reducing strategies can offer special insight into both the experience of illness and the ways out of it. Unfortunately, their personal stories are rarely promoted or represented by advocacy groups as they do not match the ‘incurable physical disease’ narrative.

## The unproven narrative of a disease with no cure can be harmful

The narrative of CFS/ME as an incomprehensible and incurable disease without any available treatment is likely to increase fear, helplessness, and loss of hope. The associated advice to rest whilst awaiting a medical cure both discourages patients from gaining control of their symptoms and creates a barrier to seeking potentially effective treatments, as well as risking worsening of symptoms and reduced quality of life. Unfortunately, this narrative is now being promoted for post-covid-19 conditions, risking a further major negative impact on public health.

## What is needed now?

We call for change. Specifically, we propose that doctors and health professionals should feel free to discuss and express different understandings of these illnesses. They should also be free to recommend any evidence-based treatments that offer a realistic hope of improvement and even recovery. In summary, we need a broader more constructive and better-informed public narrative about these disabling illnesses, if we are to make real progress in helping those who are suffering from them.
